# Mapping the Prenatal Growth of the Mandible

**DOI:** 10.1097/SCS.0000000000012468

**Published:** 2026-01-23

**Authors:** Cornelis Klop, Ruud Schreurs, Ailin M.J. Ackermans, Valerie G. Niehe, Sophie C. Visser, Bernadette S. De Bakker, Jitske W. Nolte, Alfred G. Becking

**Affiliations:** * Department of Oral and Maxillofacial Surgery, Amsterdam UMC, Academic Centre for Dentistry Amsterdam (ACTA), Amsterdam Movement Sciences, University of Amsterdam, Amsterdam; † Department of Oral and Maxillofacial Surgery 3D Lab, Radboud University Medical Centre Nijmegen, Radboud Institute for Health Sciences, Nijmegen; ‡ Department of Medical Biology, Amsterdam UMC, Amsterdam Reproduction and Development Research Institute, University of Amsterdam; § Department of Obstetrics and Gynecology, Amsterdam UMC, Amsterdam Reproduction and Development Research Institute, University of Amsterdam, Amsterdam; ‖ Department of Radiology, Groene Hart Ziekenhuis, Gouda, The Netherlands

**Keywords:** Anatomy, bone, development, embryology, fetus, human, jaw, organ size, skeleton, skull

## Abstract

**Objective:**

To establish normative data for key morphometric measurements in prenatal mandibular development.

**Methods:**

Forty-two human embryonic and fetal specimens, ranging from 8 to 37 weeks of gestational age (GA), without known abnormalities, were selected. Mandibles were segmented on histologic, micro-CT, or MDCT images. Fourteen anatomic landmarks were annotated, and key morphometric measurements were extracted, including bigonial width, bicondylar width, body length, ramus height, and gonial angle. Quadratic regression models were fitted to the measurements as a function of gestational age to assess developmental trends.

**Results:**

Mandibular ossification was first observed at Carnegie stage 19 (8+4 wk GA), adjacent to Meckel cartilage. Strong, statistically significant correlations with GA were found for all linear measurements (*R* ^2^>0.95, *P*<0.0001). The gonial angle showed a moderate but significant inverse correlation with age (*R* ^2^=0.65, *P*<0.0001).

**Conclusion:**

This study provides comprehensive normative reference data for prenatal mandibular development, spanning early embryonic to late fetal stages. The established growth curves can serve as valuable tools in prenatal diagnostics, enabling earlier and more accurate detection of craniofacial anomalies associated with syndromic and nonsyndromic mandibular malformations. The presented normative data may also aid age estimation in forensic and archeological investigations.

Together with the clavicle and maxilla, the mandible is one of the earliest bones to ossify during the primary stages of human development.^1^ With ossification first appearing in the eighth week of gestation, the mandible is derived from the mesenchyme of the ventral mandibular process of the first pharyngeal arch.^2^ The ossification of mesenchymal tissue occurs adjacent to the pre-existing Meckel cartilage.^2–4^ The ossification forms 2 separate hemimandibles, which fuse in the first or second year of life at the mandibular symphysis to form the mandible.^5,6^

Mandibular development is rather complex, mainly due to the involvement of multiple types of embryonic tissue. This makes the mandible susceptible to congenital abnormalities, some of which are associated with genetic disorders or syndromes.^7–9^ Examples of well-known anomalies that may involve the mandible are Pierre Robin sequence, Treacher Collins syndrome, and craniofacial microsomia. Pediatricians and ultrasonographers rely on knowledge of normal human development to monitor fetal growth and development and to detect congenital anomalies. In addition, the mandible is often used in fetal age estimation in archeological and forensic contexts, using metric measurements of anatomic landmarks.^10,11^

A better understanding of normal mandibular development may aid in an early and conclusive diagnosis of genetic disorders, syndromes, and malformations that involve disrupted mandibular development. Many authors have contributed to understanding the prenatal growth of the mandible.^3,4,7,8,12–27^ However, most previous research has focused on a specific age range,^7,12–14,18,22^ targeted individual anatomic structures,^13,27^ relied on limited sample sizes^12,14,18,19,21^ or did not include normative measurements.^4,16^ In this paper, we establish normative data for prenatal mandibular development.

## METHODS

### Databases

The specimens originated from 3 databases: the Carnegie Collection, the Dutch Fetal Biobank,^28^ and the forensic database of the Groene Hart Hospital. General information on the databases used in this study, such as the number of included samples, imaging modalities, and age ranges, is shown in Supplemental Table 1, Supplemental Digital Content 1, http://links.lww.com/SCS/J46.

The Carnegie Collection consists of historical specimens, collected in the period from 1910 to 1967 by the Human Developmental Anatomy Center at the National Museum of Health and Medicine (Silver Spring, MD). No ethical approval was necessary for the use of this historical collection. Histologic images of these specimens were provided by the Virtual Human Embryo Project (Louisiana State University Health Science Center, New Orleans, LA, virtualhumanembryo.lsuhsc.edu)^29^ and the 3D Embryo Atlas Project (Amsterdam UMC, University of Amsterdam, Amsterdam, the Netherlands, 3dembryoatlas.com).^30^ The histologic sections of this database were previously used by De Bakker et al^30^ to build the digital 3D Embryo Atlas. These publications also contain documentation of the year of acquisition, type of staining, and fixation medium used for these specimens, along with a detailed description of the digitizing process for the sections.

The Dutch Fetal Biobank is a donation program of embryos and fetuses after induced termination of pregnancy, spontaneous abortion, or intrauterine fetal demise (IUFD).^28^ The Dutch Fetal Biobank donation program was started in 2017 after acquiring all legal and ethical permits. Maternal written informed consent for donation to the Dutch Fetal Biobank was obtained before termination of pregnancy or delivery. The Medical Ethics Review Committee of the Amsterdam UMC granted approval of this program for research purposes (file number 2016_285). Most specimens of this database were digitized using either the Phoenix Nanotom (Baker Hughes Company, Houston, TX) or the TESCAN UniTOM XL micro-CT scanner (Tescan Orsay Holding, Brno, Czech Republic). Some older samples were imaged using a conventional Siemens SOMATOM Force CT scanner (Siemens Healthineers, Erlangen, Germany). In many cases, the biological sex of the fetus was confirmed through quantitative fluorescent-polymerase chain reaction (QF-PCR), which was also used to determine whether there were any genetic abnormalities present. The following aneuploidies were tested in this analysis: trisomy 13 (Patau syndrome), 15, 16, 18 (Edward syndrome), 21 (Down syndrome), and trisomy 22.

The forensic database of the Groene Hart Hospital (Gouda, the Netherlands) consists of images of individuals, acquired in the context of postmortem forensic investigations. The Medical Ethics Review Committee of the Amsterdam UMC also assessed the use of this database and stated that the Medical Research Involving Human Subjects Act (WMO) was not applicable (file number 2021_477). Hence, there was no additional consent required from the next of kin of the included individuals for using this data. Images of the individuals were obtained using a Toshiba Aquilion CT scanner (Toshiba Corporation, Minato, Tokyo, Japan) at the Groene Hart Hospital (Gouda, the Netherlands).

### Study Samples

Samples were included if they met the following criteria: absence of known abnormalities; presence of mandibular ossification; and gestational age (GA) and biological sex documented as accurately as possible. In total, 42 human embryos and fetuses, ranging from 8 to 37 weeks GA, were included in this study (22 male, 18 female, and 2 unknown). Five of the youngest specimens (GA: 8–10 wk, Carnegie stages 19–23) were obtained from the Carnegie Collection. The youngest specimen to be used in this study was chosen based on the first appearance of ossification of the mandible—a specimen of Carnegie stage 18 was available, but no mandibular bone could be identified. Twenty-eight specimens (GA: 9–24 wk) were provided by the Dutch Fetal Biobank. Nine specimens (GA: 31–37 wk) were obtained from the forensic database of the Groene Hart Hospital. Detailed information on the individual specimens can be found in Supplemental Table 2, Supplemental Digital Content 1, http://links.lww.com/SCS/J46.

### Segmentation

**FIGURE 1:**
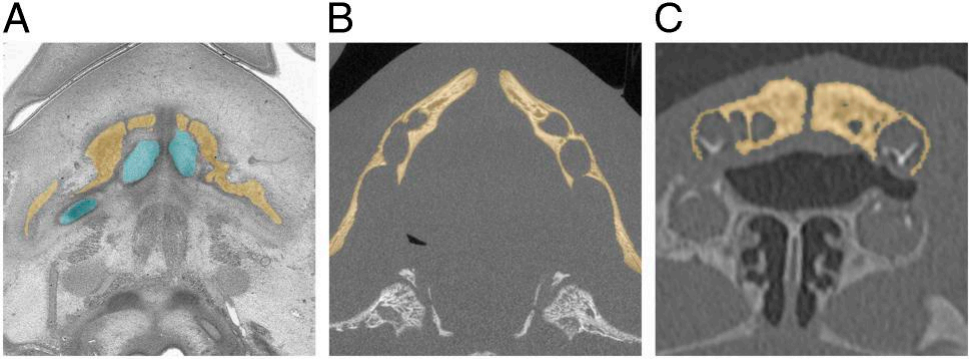
Example of segmentation of the mandible for each imaging modality. (A) Segmentation of the mandible (yellow) and Meckel cartilage (blue) on histology sections. (B) On microcomputed tomography (micro-CT) images. (C) On multidetector computed tomography (MDCT) images.

Segmentation of the mandible was performed by 1 observer using Amira3D 2022.1 (Thermo Fisher Scientific Inc., Waltham, MA). Segmentation was done using a combination of thresholding operations, interpolation, and manual delineation using a Bamboo tablet and pen (Wacom Co. Ltd, Kazo, Saitama, Japan). The segmentations were exported in stereolithography (STL) format. For the 5 specimens from the Carnegie Collection, the Meckel cartilage was also identified and exported as an STL file. An example of a segmentation for each imaging modality (histology, micro-CT, and MDCT) is shown in Figure 1A to C, respectively.

### Postprocessing

Segmentations were postprocessed in Blender 4.2 (Blender Foundation, Amsterdam, the Netherlands). First, the segmentation was split into 2 hemimandibles. Both hemimandibles were morphologically closed to fill small segmentation gaps and to reduce step artefacts due to slice thickness. This process involved extruding the object, performing voxel remeshing, projecting the result back onto the original surface by shrinkwrapping, and once more remeshing the object. Values for extrusion factors and voxel sizes were established empirically, depending on the size of the mandible.

### Landmarks and Measurements

Fourteen anatomic landmarks were annotated on the 3D models of the mandibles in Blender using an in-house developed add-on. Seven landmarks were identified on both hemimandibles, when present: the coronoid process, the buccal, lingual, posterior, and cranial points of the condyle, the gonion, and the menton. Similar anatomic landmarks were used in previous studies on mandibular growth.^31^ The landmarks were annotated to determine the developmental onset and extract basic measurements for tracking mandibular growth. The 2 bilateral menton and gonion landmarks were annotated on every sample. If a distinct gonial angle was absent, the gonion landmark was placed at the most posterior, most caudal point of the mandibular bone. Hence, the bigonial width (distance between left and right gonion landmarks) and body length (distance between gonion and menton landmarks) were calculated for all samples. The ramus height (distance between condyle and gonion landmarks) was calculated for the samples where the cranial point of the condyle was identifiable. In case the cranial part of the condyle had not fully developed yet, this point was approximated by calculating the midpoint between the buccal and lingual points of the condyle. If none of these could be identified, ramus height was not calculated. If both buccal landmarks of the condyles were present, the bicondylar width of the mandible was documented by calculating the distance between those landmarks. If the posterior point of the condyle could be identified, a vector was constructed from the gonion to this landmark. A second vector was constructed from the gonion to the menton landmark. The gonial angle was determined by calculating the arccosine of the dot product of these 2 vectors. Bilateral left and right measurements (body length, ramus height, and gonial angle) were averaged to obtain 1 value per sample. The 3D coordinates and measurements are published with open access on the following repository: zenodo.org/doi/10.5281/zenodo.16081129.

### Statistical Analysis

The measurement data were visualized against GA using graphs in MATLAB 2022b (MathWorks, Natick, MA). A quadratic function in the form of $f\left(x\right)=ax^{2}+bx+c$ was fitted to the data. The corresponding coefficients and goodness-of-fit (*R* ^2^-values) of the regression models were documented. In addition, the *P*-value comparing the fitted model to a constant model was reported for each regression, with *P-*values <0.01 considered statistically significant. A quadratic model was selected to reflect the nonlinear nature of growth over time while maintaining simplicity to reduce the risk of overfitting.

## RESULTS

### Anatomic Development

**FIGURE 2:**
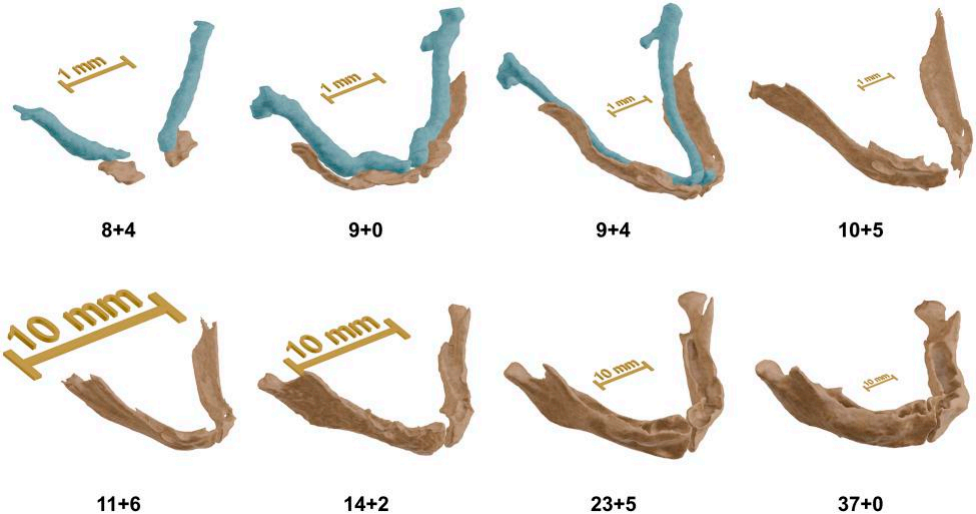
Typical appearance of the fetal mandible in various stages. Depending on the imaging modality, Meckel cartilage was also segmented (blue).

As stated, a specimen of Carnegie stage 18 was available; however, the mandibular bone was not yet present. In a sample at Carnegie stage 19 (8+4 wk GA), the first signs of mandibular ossification appeared, starting inferior and lateral to the Meckel cartilage. At this stage, there was no indication of the ramus, condylar process, or coronoid process. In samples at consecutive Carnegie stages, the ossification progressed posteriorly and cranially toward the mandibular angle, and a deep notch was seen at the inferior border of the mandibular foramen. In the first days of 9 weeks of GA, ossification occurred at the alveolar level, and a true mandibular foramen was formed. From 11 weeks GA onwards, the mandibular angle and coronoid process were recognized, and shortly after, the condylar neck. At ∼13 weeks GA, the condylar head started taking shape, which was completed around 23 weeks GA. During this period, the condylar head initially had a hollow appearance until cortical bone developed to the eventual cranial point of the condyle. By 24 weeks GA, all typical morphologic features of the mandible had been developed. From this point onwards, all structures underwent a process of consolidation and allometric growth. As a side note, the earliest detectable mineralization of the mandibular incisors was seen at 20 weeks GA. The typical appearance of the mandible at various fetal stages is demonstrated in Figure 2.

### Landmarks and Measurements

**FIGURE 3:**
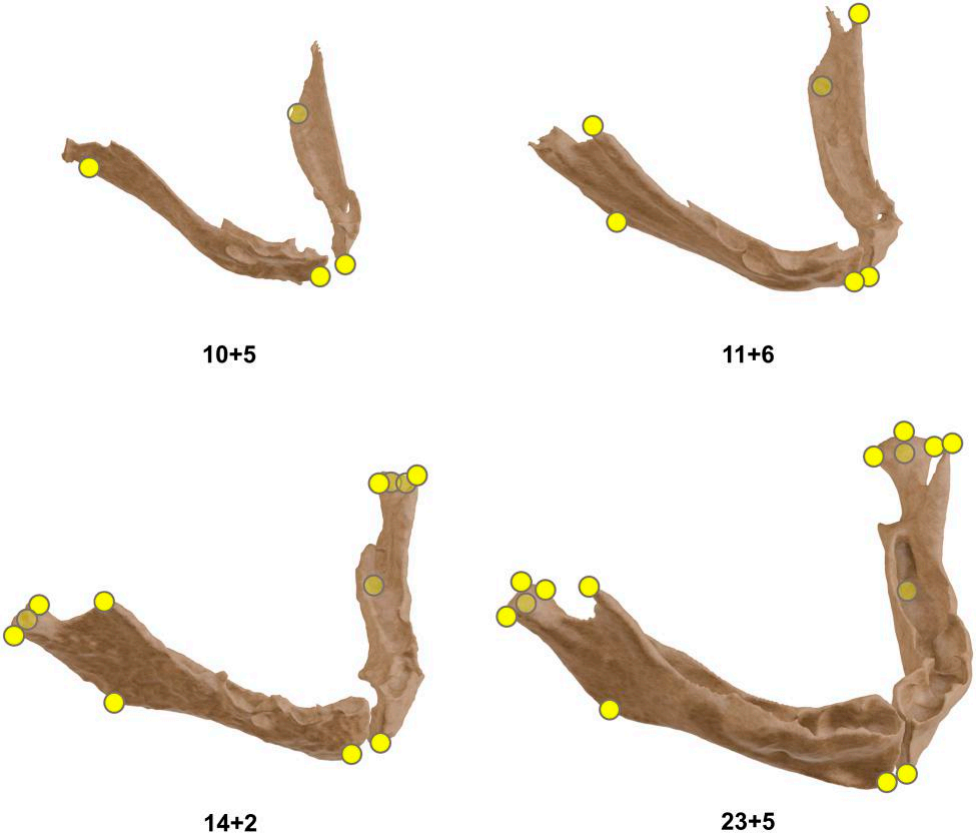
Landmarks were annotated progressively, according to their developmental onset. From 11 weeks of gestational age (GA) onwards, the coronoid processes were annotated; at ∼13 weeks of GA, the buccal, lingual, and posterior points of the condylar head were added, and the cranial point of the condyle was fully developed at around 23 weeks of GA.

**FIGURE 4:**
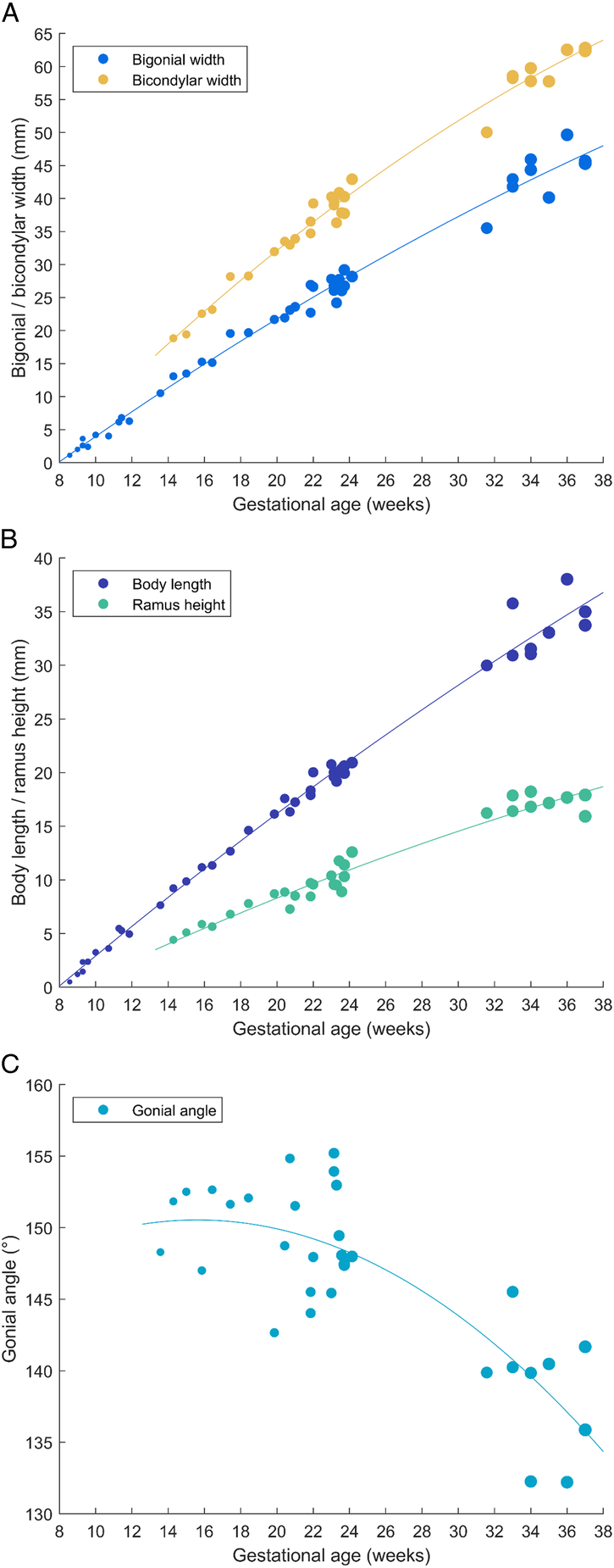
Data points and fitted regression functions for (A) Bigonial width and bicondylar width. (B) Body length and ramus height. (C) Gonial angle. Bilateral measurements (body length, ramus height, and gonial angle) were averaged between the left and right sides to obtain 1 value per sample.

Anatomic landmarks were annotated according to their appearance in mandibular development, which is demonstrated in Figure 3. For all 42 samples, the bigonial width and body length measurements were collected. The gonial angle was calculated for 32 samples, and the bicondylar width and ramus height were measured in 31 samples. Data points and fitted regression functions are plotted against GA for bigonial width and bicondylar width (Fig. 4A), body length and ramus height (Fig. 4B), and gonial angle (Fig. 4C). The equation of the functions per measurement can be found in Supplemental Table 3, Supplemental Digital Content 1, http://links.lww.com/SCS/J46. For all linear measurements, a very strong, statistically significant correlation with age (*R* ^2^>0.95, *P*<0.0001) was found, whereas the angular measurement (gonial angle) had a moderate, significant correlation with age (*R* ^2^=0.65, *P*<0.0001). Data plots and regression models were generated for male and female samples separately, but based on the available data and analysis, no differences were identified.

## DISCUSSION

The aim of this study was to provide a comprehensive overview of the prenatal development of the human mandible. This was done by imaging and segmenting the mandible on histologic, micro-CT, and MDCT images of 42 human embryos and fetuses between 8 and 37 weeks GA. This study contributes to the existing body of literature by consolidating fragmented approaches into a unified growth model.

It is generally accepted in the literature that the first signs of mandibular ossification appear at 8 weeks GA.^1,3,4,12^ In our study, ossification was first observed at Carnegie stage 19 (8+4 wk GA), thus providing more strength to previous theories. Literature describes the location of initial ossification to be lateral and inferior to Meckel cartilage,^3,12,16,27^ a finding that is confirmed by the present study. It is well known that Meckel cartilage serves as a scaffold for mandibular ossification.^2–4,12,16^ This is supported by our findings, which show that the mandibular bone develops adjacent to the cartilage, starting in the anterior region and progressing posteriorly and cranially. Also generally in agreement with our results are studies by Radlanski et al^12^ and Lee et al,^3^ who reported the onset of ossification at the condylar and coronoid process at ∼11 to 12 weeks GA. Ossification of the condylar head begins around 13 weeks GA and progresses over an extended developmental period. In our study, the superior-most point of the condyle appeared incompletely ossified until ∼23 weeks GA. This observation aligns with findings reported by Symons, who described the development of the temporomandibular joint from 9 weeks GA onwards.^27^ The exact moment of “full ossification,” however, is hard to determine and may vary depending on the interpretation of the imaging data.

Regression models for bigonial width, bicondylar width, body length, and ramus height showed strong correlations with age and statistically significant *P*-values. As most of the residuals—that is, the differences between the data points and the regression curve—were small, the presented models appear to be useful in clinical and forensic contexts. The models for body length and ramus height, in particular, had very small quadratic (*x* ^2^) coefficients, meaning they are almost as well approximated by linear functions. Many previous studies used a linear function for this reason.^7,8,12,19,21,22^ Omitting the quadratic term, however, would lead to large residuals in the early stages of development. Many earlier studies focused on a narrower age range, where a linear approximation may be adequate. Nonlinear approaches appear to yield slightly improved *R* ^2^-values, especially for broader age ranges.^14,15,24^ Given that the resulting equations remain relatively simple, we chose to retain the quadratic regression models instead of a linear approximation. The regression model for gonial angle, although statistically significant, showed only a moderate correlation with age, limiting its clinical relevance. This limitation is not particularly a major concern, as linear measurements are typically easier to obtain than angular measurements in clinical practice (eg, using ultrasound). In the present study, the gonial angle demonstrated a decreasing trend as a function of gestational age. This is consistent with several earlier studies^3,19–21^ and with the decreasing trend of the gonial angle in the postnatal phase.^31,32^ However, the fetal development of the gonial angle remains controversial, as other studies have reported no significant change^25^ or even an increasing trend in later fetal stages.^17^ These discrepancies may result from differences in study samples, imaging modalities, or measurement techniques. Most mandibles (40/42) in our study were classified by biological sex, but separate plots for male and female samples failed to reveal consistent differences, presumably due to the sample size and variation within both groups relative to the broad age range.

Compared with previous studies focusing on isolated gestational stages or specific anatomic features, our data provide continuous normative reference values spanning early embryonic to late fetal development. The study was conducted with a considerable sample size, which was distributed relatively evenly over the age range. No samples were available for the gestational period between 25 and 31 weeks. This is mainly due to the Dutch legislation, which permits induced termination of pregnancy up to 24 weeks GA. However, as all typical structures of the mandible have already been developed by the 24th week, the measurements reported in this study can be interpolated for this interval. Three different imaging modalities were used, but the technique and resolution of each modality were appropriate for the size and age of the respective samples. None of the samples suffered from an anatomically identifiable cause of death or any visible abnormalities. Yet some causes of pregnancy termination, such as spontaneous abortion and IUFD, suggest a possible underlying pathology in the embryo or fetus. For over half of the samples, QF-PCR was hence used to confirm the absence of the most common genetic abnormalities. The techniques used in this study, particularly image segmentation and landmark annotation, were validated in a previous study,^31^ albeit in postnatal mandibles.

The authors would have ideally developed a 3-dimensional growth model of the prenatal mandible, similar to an earlier presented growth model of the postnatal phase.^31^ However, for classic statistical shape modeling, all included models are required to have a semantically similar shape. The key difficulty with studying prenatal growth is establishing correspondence across developmental stages, where anatomy is incomplete or evolving. Standard template-based registration methods assume that all structures are present in every target, which fails in this case. In the future, advanced techniques, such as partial shape modeling or region-based correspondence with dynamic topology, may be employed to capture the true 3-dimensional shape of the prenatal mandible. Instead of a 3-dimensional growth model, normative measurements were presented in this study, which can be directly translated to a clinical setting. Besides clinical relevance, these data may also be useful to estimate age in archeological or forensic contexts. Fetal age estimation in these contexts is often based on a small number of ossified dry bone specimens from archeological populations, though some studies using radiology have been performed.^10,11^ The normative data presented in this study may serve as a valuable reference for clinicians during prenatal screening, and for forensic and archeological applications involving age estimation.

## Supplementary Material
