# Supplementary material for: Mapping the Prenatal Growth of the Mandible
Source: J Craniofac Surg. 2026 Jan 23;37(6):1513–7. doi: 10.1097/SCS.0000000000012468 (PMC13200890; doi:10.1097/SCS.0000000000012468)
Supplement: Supplementary file 1 [file scs-37-1513-s001.docx]

| **Supplemental** **Table 1:** Information on the imaging databases. | | | | | | |
| --- | --- | --- | --- | --- | --- | --- |
| **Database** | **Primary imaging modality** | **No. of samples** | **GA (weeks)** | **Pixel size* (mm)** | **Z-resolution (mm)** | **Medical ethics review** |
| Carnegie collection | Histology | 5 | 8 - 10 | 0.005 – 0.025 | 0.040 – 0.150 | N/A |
| Dutch Fetal Biobank | MicroCT | 28 | 9 - 24 | 0.022 – 0.078 | 0.022 – 0.078 | Amsterdam UMC MERC file # 2016_285 |
| Groene Hart Hospital | MDCT | 9 | 31 - 37 | 0.50 | 0.21 – 0.59 | Amsterdam UMC MERC file # 2021_477 |
| MERC = medical ethics review committee, (MD)CT = (multi-detector) computed tomography *square pixels, proportional in both directions | | | | | | |

| **Supplemental Table 2:** Detailed information on the specimens, sorted by gestational age. | | | | | | |
| --- | --- | --- | --- | --- | --- | --- |
| **Specimen** | **Database** | **Imaging modality** | **GA (weeks + days)** | **Indication for termination of pregnancy** | **Anatomically identifiable cause of death / abnormalities (*QF-PCR confirmed)** | **Sex (*QF-PCR confirmed)** |
| 1 | CC | Histology | 8 + 4 | Hysterectomy | None | F |
| 2 | CC | Histology | 9 + 0 | Spontaneous abortion | None | M |
| 3 | DFB | microCT | 9 + 2 | Social | None* | M* |
| 4 | CC | Histology | 9 + 2 | Hysterectomy | None | M |
| 5 | CC | Histology | 9 + 4 | Hysterectomy | None | F |
| 6 | CC | Histology | 10 + 0 | Ectopic pregnancy | None | M |
| 7 | DFB | microCT | 10 + 5 | Maternal | None | N/A |
| 8 | DFB | microCT | 11 + 2 | Social | None | M |
| 9 | DFB | microCT | 11 + 3 | Social | None* | F* |
| 10 | DFB | microCT | 11 + 6 | Social | None* | F* |
| 11 | DFB | microCT | 13 + 4 | Spontaneous abortion | None* | M* |
| 12 | DFB | microCT | 14 + 2 | Placenta percreta | None | N/A |
| 13 | DFB | microCT | 15 + 0 | Spontaneous abortion | None | F |
| 14 | DFB | microCT | 15 + 6 | Spontaneous abortion | None* | F* |
| 15 | DFB | microCT | 16 + 3 | Spontaneous abortion | None* | F* |
| 16 | DFB | microCT | 17 + 3 | Spontaneous abortion | None | F |
| 17 | DFB | microCT | 18 + 3 | Spontaneous abortion | None* | M* |
| 18 | DFB | MDCT | 19 + 6 | Spontaneous abortion | None* | M* |
| 19 | DFB | microCT | 20 + 3 | Social | None* | M* |
| 20 | DFB | microCT | 20 + 5 | IUFD | None* | M* |
| 21 | DFB | microCT | 21 + 0 | Social | None* | M* |
| 22 | DFB | microCT | 21 + 6 | Social | None* | M* |
| 23 | DFB | microCT | 21 + 6 | IUFD | None* | F* |
| 24 | DFB | microCT | 22 + 0 | Social | None* | F* |
| 25 | DFB | microCT | 23 + 0 | Social | None* | F* |
| 26 | DFB | microCT | 23 + 1 | Social | None* | M* |
| 27 | DFB | microCT | 23 + 1 | Social | None* | M* |
| 28 | DFB | microCT | 23 + 2 | IUFD | None* | M* |
| 29 | DFB | MDCT | 23 + 3 | Social | None* | M* |
| 30 | DFB | microCT | 23 + 4 | Social | None* | F* |
| 31 | DFB | microCT | 23 + 5 | Social | None* | F* |
| 32 | DFB | microCT | 23 + 5 | Social | None* | F* |
| 33 | DFB | microCT | 24 + 1 | Social | None* | F* |
| 34 | GHH | MDCT | 31 + 4 | IUFD | None | M |
| 35 | GHH | MDCT | 33 + 0 | Maternal | None | M |
| 36 | GHH | MDCT | 33 + 0 | Unknown | None | F |
| 37 | GHH | MDCT | 34 + 0 | Unknown | None | M |
| 38 | GHH | MDCT | 34 + 0 | IUFD | None | F |
| 39 | GHH | MDCT | 35 + 0 | Unknown | None | M |
| 40 | GHH | MDCT | 36 + 0 | Maternal | None | M |
| 41 | GHH | MDCT | 37 + 0 | Social | None | M |
| 42 | GHH | MDCT | 37 + 0 | Unknown | None | F |
| GA = gestational age, CC = Carnegie collection, DFB = Dutch Fetal Biobank, GHH = Groene Hart Hospital, IUFD = intrauterine fetal demise, QF-PCR = quantitative fluorescent-polymerase chain reaction, (MD)CT = (multi-detector) computed tomography, M = male, F = female, N/A = not available | | | | | | |

| **Supplemental** **Table 3:** Quadratic regression equations fitted to the data points per measurement, including the R-squared-value (coefficient of determination, indicating goodness-of-fit) and p-value from the F-test comparing the fitted model to a constant (intercept-only) model. | | | |
| --- | --- | --- | --- |
| **Measurement ^1,2^** | **Formula ^3,4^** | **R-squared** | **p-value** |
| $bigonial width$ | $-0.012 {age}^{2}+2.13 age-16.16$ | 0.99 | <0.0001 |
| $bicondylar width$ | $-0.024 {age}^{2}+3.18 age-21.71$ | 0.99 | <0.0001 |
| $body length$ | $-0.0065 {age}^{2}+1.52 age-11.64$ | 0.99 | <0.0001 |
| $ramus height$ | $-0.0057 {age}^{2}+0.91 age-7.57$ | 0.95 | <0.0001 |
| $gonial angle$ | $-0.032 {age}^{2}+1.01 age+142.67$ | 0.65 | <0.0001 |
| ^1^ output distances (width, length, height) in mm  ^2^ output angles in degrees  ^3^ input gestational age in weeks  ^4^ formulas only apply to evaluated age range, see Figure 4 | | | |
